# Effect of Acid Whey Pretreatment Using Ultrasonic Disintegration on the Removal of Organic Compounds and Anaerobic Digestion Efficiency

**DOI:** 10.3390/ijerph191811362

**Published:** 2022-09-09

**Authors:** Joanna Kazimierowicz, Marcin Zieliński, Izabela Bartkowska, Marcin Dębowski

**Affiliations:** 1 Department of Water Supply and Sewage Systems, Faculty of Civil Engineering and Environmental Sciences, Bialystok University of Technology, 15-351 Bialystok, Poland; 2 Department of Environmental Engineering, Faculty of Geoengineering, University of Warmia and Mazury in Olsztyn, 10-720 Olsztyn, Poland

**Keywords:** acid whey, ultrasonic disintegration, methane fermentation, biogas, pretreatment, bacterial community

## Abstract

Acid whey is a by-product of the dairy industry that should be utilized or appropriately neutralized. Anaerobic processes represent a group of prospective methods for whey processing, and a key priority in their development is to improve their technological and economical effectiveness. The present study aimed to determine the effect of ultrasonic disintegration (UD) of acid whey on the course and effectiveness of methane fermentation. The study results demonstrated that extending the UD duration resulted in increased concentrations of dissolved forms of COD and TOC, efficiency of organic matter biodegradation, and CH_4_ production. The best effects were achieved at 900 s US, including CH_4_ production of 0.203 ± 0.01 dm^3^/gCOD_in._ and CH_4_ content accounting for 70.9 ± 2.8%. Organic compounds were removed with the following efficiencies: COD—78.7 ± 2.1%, TOC—80.2 ± 1.3%, and BOD_5_—84.1 ± 1.6%. The highest net energy gain of 5.763 Wh was achieved upon UD of 300 s. Extension of UD time had no significant effect on the improvement in the energetic effectiveness of anaerobic digestion. A strong positive correlation was found between COD and TOC concentrations in the dissolved phase and CH_4_ production yield.

## 1. Introduction

Acid whey is a by-product of dairy processing, including cheese-making or casein production [1]. It is estimated that the volume of whey obtained ranges from 0.8 to 0.9 L per 1 L of processed milk [2], whereas its annual global production is estimated at 180–190 × 10^6^ tons [3]. The EU member states annually produce 40 × 10^6^ tons of cheese whey, and the annual whey surplus is approximately 13 × 10^6^ tons [4]. In many cases, this by-product can be processed and used as a dietary supplement or additive to feedstuffs or confectionery products, and also to produce cheeses or drinks containing whey protein and finally to enhance the texture of meat and poultry products [5,6]. Avenues of acid whey applications are determined by its quality, primarily including contents of lactose, soluble proteins, fats, and mineral salts [7]. Inadequate quality of this waste necessitates its neutralization and management, whereas inappropriately processed whey poses a serious threat to the quality of the natural environment [8]. This threat is due to the high loads of organic compounds in whey [9], namely COD with concentrations ranging from 50 to 102 kg/m^3^, and those of BOD_5_ ranging from 27 to 60 kg/m^3^ [10,11,12]. Whey also contains fats and proteins (0.99–10.58 kg/m^3^ and 1.4–8.0 kg/m^3^, respectively [11]), lactose (45–50 kg/m^3^), mineral salts (8–10% of dry extract) [13], as well as nitrogen (0.2–1.76 kg/m^3^) and phosphorus (0.124–0.54 kg/m^3^) compounds, which pose a severe risk of eutrophication, making direct whey management in the natural environment impossible [11]. Whey has also high concentrations of lactic acid (0.5 kg/m^3^) and citric acid, as well as nonprotein nitrogen compounds (urea and uric acid) and B-group vitamins [14].

Whey may be utilized in aerobic wastewater treatment systems operating based on the activated sludge method [15]. Though effective in reducing the concentrations of organic compounds and the removal of biogenes, as claimed by many authors, this technology should not be harnessed for the biodegradation of whey contaminants due to its multiple drawbacks [16]. Aerobic systems are highly energy-consuming and operate best at low organic compound loads, which directly contributes to their large cubatures and occupied spaces, whereas their operation generates high amounts of hardly degradable sewage sludge [17].

A competition and a justified alternative to the activated sludge technology are offered by methods based on anaerobic processes, including hydrogen and methane fermentation [18]. They are in line with generally applicable assumptions of bioeconomy, energy recycling, and circular economy and support the need to develop renewable energy sources and reduce carbon dioxide emissions [19]. Anaerobic biodegradation of whey results in the production of biogas having high concentrations of hydrogen and methane and the generation of fermented sludge, which is deemed to be a valuable organic fertilizer [20]. Due to the thorough knowledge on methane fermentation technology and its widespread use on a large scale, its implementation seems to be particularly attractive in the process of anaerobic whey biodegradation [21].

Pretreatment methods are increasingly often used to improve the technological effectiveness of methane fermentation, as they allow enhancing biodegradability, increase the production of gaseous metabolites of anaerobic bacteria and methane concentration, enable an increase in the load of organic compounds, and reduce the hydraulic retention time in the technological system [22]. This allows reducing the total volume of the reactors and surface area needed for their location, reduces investment costs, and may lead to an improvement in the energy balance and ultimate economic effect [23].

Ozone treatment, dilution, membrane filtration, lactic acidification and neutralization with lime, as well as microwave radiation (MR) have been harnessed to improve the efficiency of methane fermentation of whey in the research and exploitation works carried out so far [24,25,26]. In contrast, there is a lack of reports on the impact of ultrasonic disintegration pretreatment on the course of the methane fermentation process of this organic substrate. Studies in this area were conducted by Mainardis et al. (2019) [27]. The results they obtained were promising but require verification based on a greater load of experimental data.

Ultrasounds are acoustic waves with frequencies above 16 kHz, widely used in medical ultrasonography, cosmetology, and industry [28]. They elicit thermal and nonthermal effects; the first occurs when the energy absorbed by matter is converted into heat, whereas the other one can be classified as cavitation and stress mechanisms [29]. The application of ultrasonic disintegration for whey pretreatment is justified by the wide application possibilities of this technical solution for other processes and pollutants, as presented in many publications [30]. Ultrasounds have been used in processes of water disinfection [31], to enhance the susceptibility of an organic contaminant to biodegradation [32], to remove ammonia from wastewater [33], to pretreat sewage sludge before fermentation or dehydration [34], to aid membrane filtration [35], etc. [36].

This study was undertaken to determine the impact of deploying ultrasonic disintegration (UD) of acid whey prior to methane fermentation on the removal efficiency of organic contaminants as well as the volume and qualitative composition of biogas produced. It focused on adjusting the load of ultrasound energy that would ensure the highest rate of biochemical anaerobic conversion, the highest concentration of methane produced, and a positive energy balance of the entire process.

## 2. Materials and Methods

### 2.1. Experimental Design

In this study, the impact of UD on the efficiency of methane fermentation of acid whey was performed in five experimental series differing in the energy load supplied to the substrate. The load of ultrasound energy was determined by the time of whey exposure to sonication. Table 1 presents the experimental design, UD duration, and energy inputs into the system used in consecutive experimental series.

### 2.2. Materials

Experiments were conducted using the solutions prepared from acid whey powder (Blattin, Poland). Whey powder (100 g) was added to 1 dm^3^ of tap water and mixed with a three-blade vertical mechanical stirrer (Microstar 7.5 Digital, IKA Werke GmbH & Co. KG, Staufen, Germany) at 150 rpm for 30 min to allow the whey to dissolve. The acid whey solution was pretreated into a UP400 s ultrasound disintegrator (Hielscher Ultrasonics GmbH, Teltow, Germany) and then fermented in respirometers (WTW GmbH, Weilheim, Germany). Table 2 presents the characteristics of the acid whey solution used in the study. 

Sewage sludge was used as the inoculum in respirometric tanks derived from closed fermentation tanks of the municipal wastewater treatment plant in Olsztyn (Poland). The tanks operated at an organic load rate (OLR) approximating 2.5 kg o.d.m./m^3^·d, hydraulic retention time (HRT) of 20 days, under conditions of mesophilic fermentation at a temperature (T) of (T) 35 °C. Afterward, the sewage sludge was adapted to acid whey degradation in reactors operating in a continuous mode for 30 days (OLR = 5.0 kg COD/m^3^·d, HRT = 48 h, T = 35 °C). Table 3 presents the characteristics of the sewage sludge used in the experiments.

### 2.3. Laboratory Equipment

**Figure 1:**
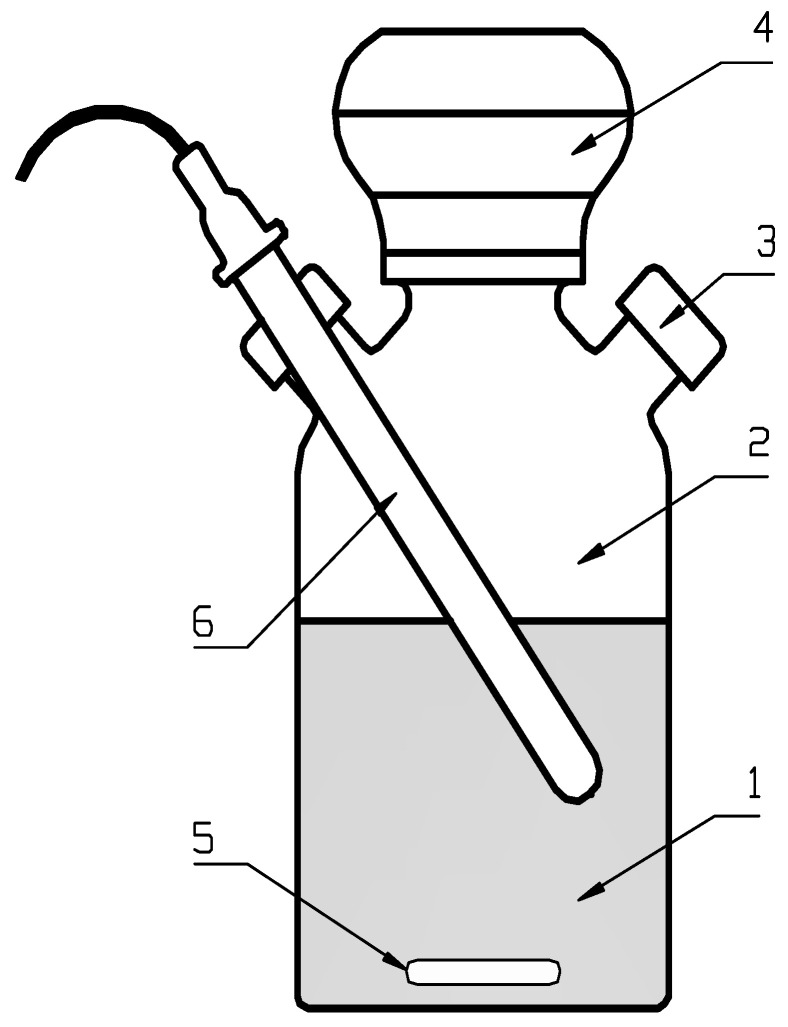
Single respirometric measuring kit, 1—measuring tank liquid phase, 2—measuring tank gaseous phase, 3—side port for biogas collection, 4—measuring and registering device, 5—magnetic stirrer, and 6—pH electrode.

UD of acid whey was performed by means of a UP 400 S ultrasound disintegrator (Hielscher Ultrasonics GmbH, Teltow, Germany), operating at a power of 400 W and emitting sounds with a frequency of 24 kHz. The time of whey exposure to ultrasounds varied in the successive experimental series, whereas the volume and concentration of the disintegrated whey solution remained stable. The UD process goal was to analyze whey susceptibility to anaerobic degradation. This goal was achieved by respirometric measurements (WTW GmbH, Weilheim, Germany). The total volume of respirometers was 2.0 dm^3^ (Figure 1).

Respirometers registered partial pressure changes in the measuring tank triggered by biogas production. Anaerobic sludge (1000 cm^3^) was fed into reaction tanks, and then the assumed volume of acid whey solution was dosed in, namely the whey solution with the COD concentration of 100,000 ± 1200 gO_2_/dm^3^ was fed in a single dose of 50 cm^3^. This allowed achieving the initial reactor load of 5 g COD/dm^3^. Simultaneously, measurements were conducted for the blank sample by determining the respirometric activity of the anaerobic sludge not fed with the organic substrate. To ensure anaerobic conditions inside fermentation tanks, before the measurements, their contents were purged with nitrogen for 5 min with an efficiency of 150 dm^3^/h. A complete measuring kit was placed in a thermostatic cabinet with the hysteresis not exceeding ±0.5 °C. Measurements were carried out at a temperature of 35 °C for 20 days. Pressure values in the reaction tank were registered every 6 h.

### 2.4. Analytical Measurements

The acid whey was determined for the contents of chemical oxygen demand (COD), total phosphorus (P_tot._), orthophosphate (P–PO_4_), total nitrogen (N_tot._), and ammonia nitrogen (N–NH_4_) using a DR 5000 spectrophotometer with an HT 200 s mineralizer (Hach-Lange GmbH, Düsseldorf, Germany). Biochemical oxygen demand (BOD_5_) was determined according to PN-EN 1899-1. The TOC content was determined by means of a TOC-L analyzer TOC-L (Shimadzu, Kyoto, Japan). In turn, the contents of COD and TOC in the dissolved phase were determined after acid whey solution filtration using a kit for membrane vacuum filtration (MVF) consisting of Advantec with a PTFE hydrophilic membrane filter (Advantec MFS, Inc., Dublin, CA, USA) mounted on an MBS 1 filtration kit (Whatman International Ltd., Maidstone, UK) using a Mobile 20 (DILO Company, Inc., Odessa, FL, USA) vacuum pump. The filter had a pore size of 0.20 µm, porosity of 71%, and thickness of 35 µm. The bubble point was ≥0.38 MPa, and the flow rate was 14 cm^3^/min/cm^2^. These filters are resistant to chemicals and pH, and their maximum working temperature is 100 °C. The temperature in the acid whey after ultrasonic disintegration was measured using a Greisinger TEMP 2 (Regenstauf, Germany) temperature meter (−200 ± 850 °C) equipped with a Pt100 sensor.

The contents of dry matter, organic dry matter, and mineral dry matter in the anaerobic sludge were determined with the gravimetric method. Biomass samples dried at 105 °C were determined for the contents of total carbon (TC), total organic carbon (TOC), and total nitrogen (N_tot_) (Flash 2000 analyzer, Thermo Scientific, Waltham, MA, USA). The content of total phosphorus (P_tot_) was determined with the colorimetric method with ammonium metavanadate (V) and ammonium molybdate after sample mineralization in a mixture of sulfuric (VI) and chloric (VII) acids at a wavelength of 390 nm using a DR 2800 spectrophotometer (Hach-Lange GmbH, Düsseldorf, Germany). The pH value of H_2_O was potentiometrically measured.

The methane content of the biogas was analyzed using an Agillent 7890 A gas chromatograph (GC) (Santa Clara, CA, USA). The volume of the biogas produced, expressed per normal conditions, was computed based on the pressure changes in the measuring tank. Respirometric studies also included the determination of the biogas production rate (r) depending on the experimental variant.

Consortia of anaerobic microorganisms were analyzed at the end of the experiment following the FISH procedure. Four molecular probes were used for hybridization: bacteria-universal probe EUB338 [37], archaea-universal probe ARC915 [38], *Methanosarcinaceae*-specific probe MSMX860, and *Methanosaeta*-specific probe MX825 [39]. The samples were analyzed in an epifluorescent microscope (100 objective, total enlargement ×1000) (Nikon Eclipse, Nikon, Tokyo, Japan). The population numbers of the selected bacterial groups were estimated and compared with the cells stained with DAPI by means of Image J software [40].

### 2.5. Computation Methods

The specific energy input (E_s_) was calculated using Equation (1):

E_s_ = P_D_ × T_D_ × V_W_ (1)

where

P_D_—disintegrator power (W);

T_D_—disintegration duration (h);

V_W_—volume of whey introduced (dm^3^). 

The energy output (E_out_) generated from methane production was calculated using the following equation:

E_out_ = Y_CH4_ × CV_CH4_ × M_COD_ [Wh] (2)

where

Y_CH4_—methane yield (dm^3^/g COD);

CV_CH4_—methane calorific value (Wh/dm^3^);

M_COD_—COD mass (g COD). 

The net energy gain (E_net_) was calculated as follows (3):

E_net_ = E_out_ − E_S_ [Wh] (3)

The unit specific energy input (E_Us_) per COD_in._ mass load was calculated using the following equation:

E_Us_ = E_s_/ M_COD_ [Wh/g COD] (4)

where

E_Us_—unit specific energy input (Wh/g COD);

E_s_—specific energy input (Wh);

M_COD_—COD mass (g COD). 

The unit energy output (E_Uout_) obtained per COD_in._ mass load was calculated using the following equation:

E_Uout_ = E_out_/ M_COD_ [Wh/g COD] (5)

where

E_Uou_—unit energy output (Wh/g COD);

E_out_—energy output (Wh);

M_COD_—COD mass (g COD). 

The unit net energy gain (E_Unet_) was calculated as follows:

E_Unet_ = E_Uout_ − E_Us_ [Wh/g COD] (6)

### 2.6. Statistical Analysis

Experiments were performed in four replicates. The statistical analysis of the experimental results was carried out using a STATISTICA 13.1 PL package (StatSoft, Inc., Tulsa, OK, USA). One-way analysis of variance (ANOVA) was performed to determine the significance of the differences between variables. The Tukey (HSD) test was applied to determine the significance of the differences between the analyzed variables. In the tests, results were considered significant at *p* = 0.05.

## 3. Results and Discussion

### 3.1. Changes in the Concentration of Organic Compounds in the Dissolved Phase

The analysis of changes in the concentration of organic compounds in the dissolved phase represents an intermediate method that aids in the evaluation of the impact of organic substrate pretreatment techniques applied before anaerobic digestion [41]. The pretreatment’s goal is to damage compact organic structures, disintegrate them, and accelerate their transfer to the liquid phase, thereby increasing substrate availability to anaerobic bacteria, including mainly the hydrolyzing and acidogenic ones [42]. Efficient conversion of organic compounds to volatile fatty acids directly affects the rate and effectiveness of methanogenesis and improves digestate stability [43]. The most frequently monitored intermediate indicators of the efficiency of pretreatment methods tested include concentrations of COD, TOC, glucose, and other sugars in the dissolved phase [44].

The present study analyzed changes in the concentrations of dissolved COD and TOC depending on the duration of acid whey ultrasonic disintegration. Analogous tendencies of changes were observed in the case of both of these indicators. The greatest difference was obtained between the control series and the series with 300 s UD. The concentration of COD_diss._ increased by 11,700 ± 530 mgO_2_/dm^3^, whereas that of TOC_diss._ by 13,200 ± 442 mg/dm^3^ (Table 4). The increases in COD_diss._ and TOC_diss._ caused by disintegration time extension in series 2–4 were not as that dynamic; however, the differences observed between the subsequent series proved to be statistically significant. In series 4, the concentration of COD_diss._ was 52,300 ± 645 mgO_2_/dm^3^, whereas that of TOC_diss._ was 39,800 ± 583 mg/dm^3^ (Table 4). Ultrasonic disintegration spanning for 1200 s failed to cause statistically significant technological effects. The concentrations of the aforementioned indicators obtained in series 5 were 53,600 ± 691 mgO_2_/dm^3^ and 40,200 ± 564 mg/dm^3^, respectively.

The increase in the COD_diss._ concentration caused in various types of wastewater upon UD has been confirmed in ample previous studies [45,46]. Mainardis et al. (2019) found no correlations between the input energy of UD and COD_diss._ concentration in whey [27]. The concentration of COD_diss._ increased in skimmed whey by 14.5%, whereas only UD performed at 80 W caused a barely 2% increase in COD_diss._ in full-fat whey [27]. Sangave and Pandit (2004) [32] harnessed low-frequency ultrasounds (22 kHz, 120 W) for the pretreatment of distillery wastewater and concluded that this pretreatment had no effect on the changes in COD_diss._ concentration. However, they observed that UD increased the biodegradability of wastewater by converting their molecules into simpler forms [32].

### 3.2. Biogas Production and Methane Content of Biogas

**Figure 2:**
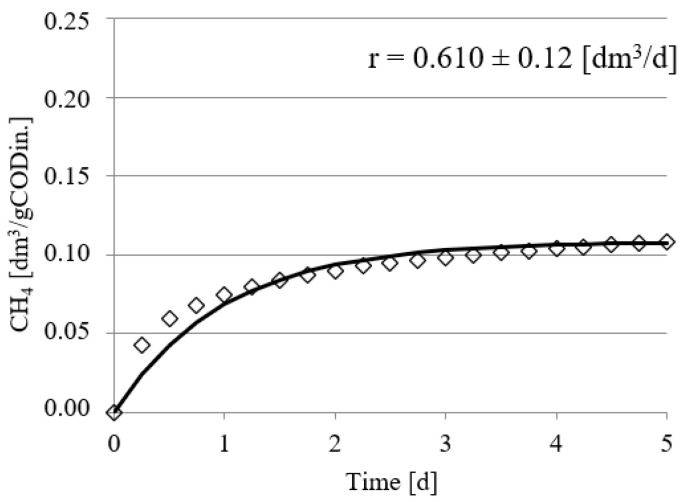
Course of methane fermentation in series 1.

**Figure 3:**
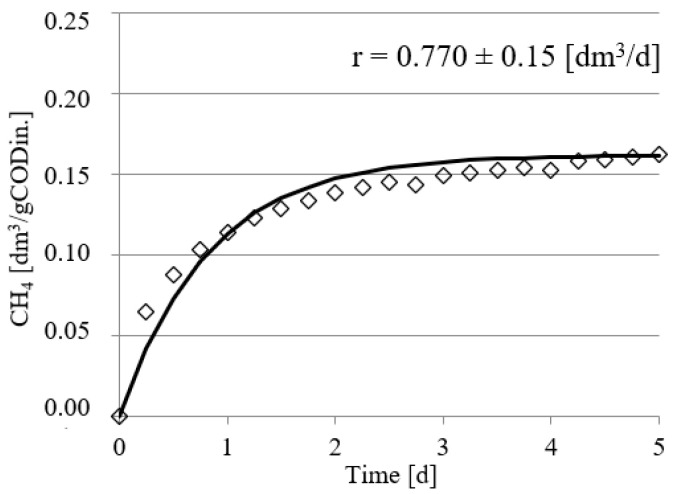
Course of methane fermentation in series 2.

**Figure 4:**
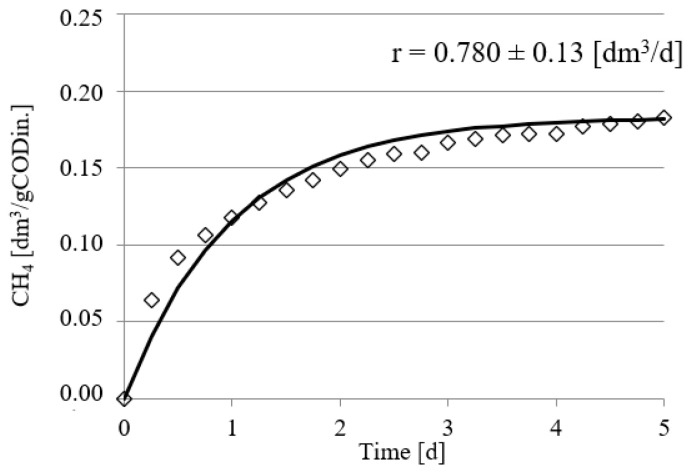
Course of methane fermentation in series 3.

**Figure 5:**
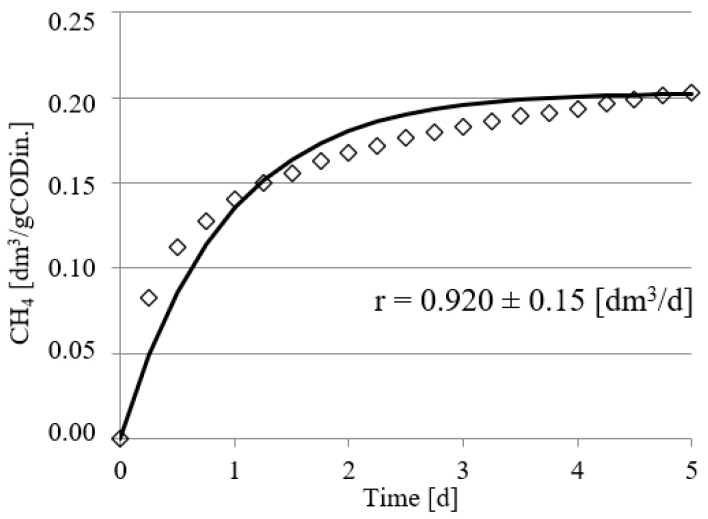
Course of methane fermentation in series 4.

**Figure 6:**
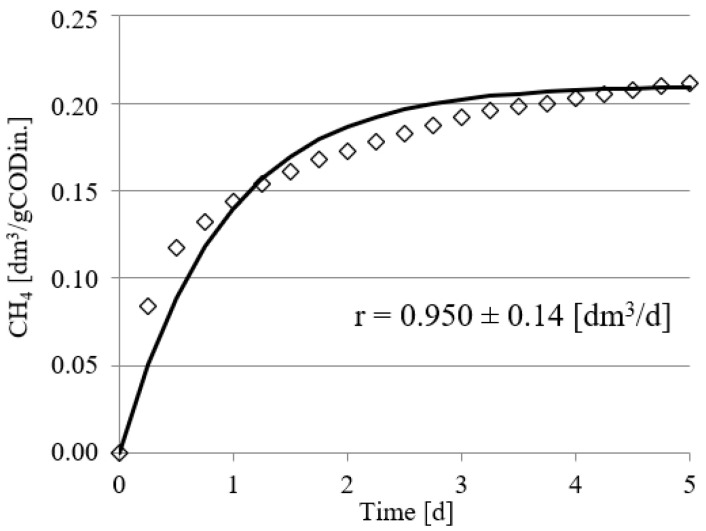
Course of methane fermentation in series 5.

**Figure 7:**
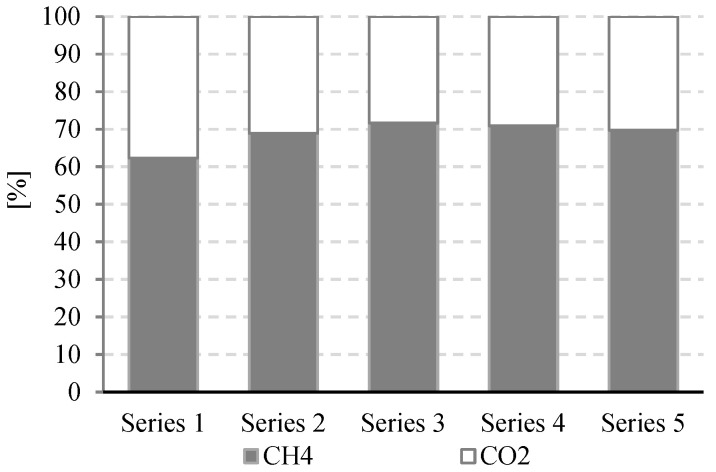
Methane content of biogas in particular experimental series.

Experimental works performed in this study confirmed the positive impact of UD on the course of methane fermentation of acid whey. Statistically significant differences were observed in the volume CH_4_ produced and in its production rate. In the control series (series 1), the CH_4_ production rate (r) was at r = 0.610 ± 0.12 dm^3^/d, and its volume by unit produced was 0.108 ± 0.11 dm^3^ CH_4_/gCOD_in._ (Figure 2). The UD performed at 300 s in series 2 ensured biogas production of 0.162 ± 0.15 dm^3^ CH_4_/gCOD_in._, which corresponded to the total volume of CH_4_ reaching 0.810 ± dm^3^. In this experimental series, the rate of CH_4_ production was at r = 0.770 ± 0.15 dm^3^/d (Figure 3). The above values were significantly higher compared with those noted in series 1 (control). Extension of UD duration to 600 s contributed to CH_4_ production increase to 0.183 ± 0.11 dm^3^ CH_4_/gCOD_in._ and its production rate increase to r = 0.780 ± 0.13 dm^3^/d (Figure 4). The highest efficiency of methane fermentation was determined in series 4 and 5, wherein UD spanned for 900 s and 1200 s, respectively. In both these series, the CH_4_ production did not significantly differ, as it reached 0.203 ± 0.15 dm^3^ CH_4_/gCOD_in._ in series 4 (Figure 5) and 0.211 ± 0.12 dm^3^ CH_4_/gCOD_in._ in series 5 (Figure 6). The total volumes of CH_4_ produced were at 1.015 ± 0.16 dm^3^ CH_4_ (r = 0.920 ± 0.15 dm^3^/d) (Figure 5) and 1.055 ± 0.15 dm^3^ CH_4_/gCOD_in__._ (r = 0.950 ± 0.14 dm^3^/d), respectively (Figure 6). The lowest CH_4_ concentration in the biogas, reaching 62.3 ± 2.1%, was determined in the control series (Figure 7). The ramp increase in the concentration of this biogas component to 68.9 ± 3.5% was noted in series 2 (Figure 7). In the remaining experimental series, the CH_4_ content was equal and fitted within a narrow range from 69.7 ± 1.7% in series 5 to 71.6 ± 3.2% in series 3 (Figure 7).

In the study by Mainardis et al. (2019), the ultrasound disintegration of whey ensured a much higher methane production at 507.9 cm^3^ CH_4_/gVS_added_, which indicates a methane yield gain by 16% compared with the nonpretreated whey (437.3 cm^3^ CH_4_/gVS_added_) [27]. The best effects were achieved at the ultrasonic disintegrator power of 40 W and disintegration time of 300 s. Contrary to the studies presented in this manuscript, the successive increase in sonication energy, applied either for a longer period of time (600 s) or at a higher power of 80 W, was not reflected in the expected increase in the methane yield [27]. These results demonstrated that extending UD duration resulted in increased CH_4_ production. The best effects were achieved at 900 s US, including CH_4_ production of 0.203 ± 0.01 dm^3^/gCOD_in._ and CH_4_ content accounting for 70.9 ± 2.8%. Gannoun et al. (2008) pretreated whey using predigestion by *L. paracasei*. This treatment ensured biogas production at 280–380 dm^3^ biogas/kgCOD removal at the OLR of 3 gCOD/dm^3^ d [24]. In turn, Skripsts et al. (2011) deployed ozonation as a whey pretreatment method [26]. They produced 696.1 dm^3^/kgVS of biogas with methane content of 62.3%. Interestingly, significantly higher values were reported in the control sample, i.e., 732.1 dm^3^/kgVS biogas with methane content of 61.4% [26]. The percentage of methane was significantly lower than that obtained in this manuscript (maximally 71.6 ± 3.2% in series 3).

### 3.3. Course of pH Changes

**Figure 8:**
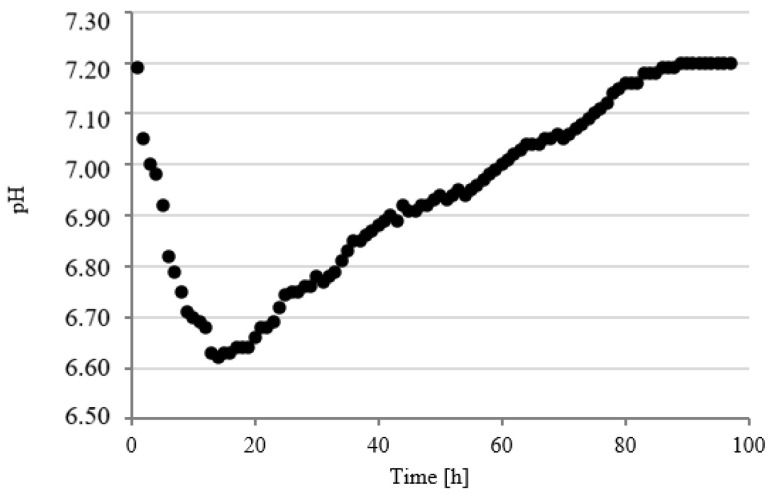
Course of pH changes in series 1.

Changes in pH values were continuously analyzed in model respirometric tanks throughout the experimental works. The courses of curves characterizing the pH value were similar regardless of the experimental series. In the first series, the value of this parameter decreased to pH 6.61 ± 0.12 within the first 14 h of fermentation and then successively and slowly increased to pH 7.20 ± 0.15 after 90 h (Figure 8).

**Figure 9:**
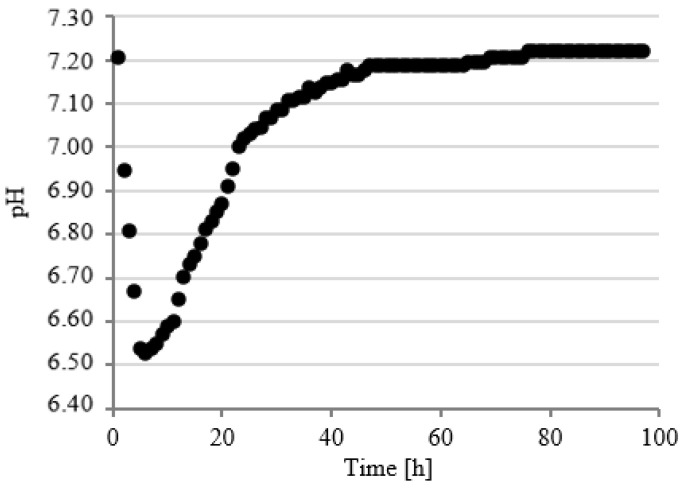
Course of pH changes in series 2.

**Figure 10:**
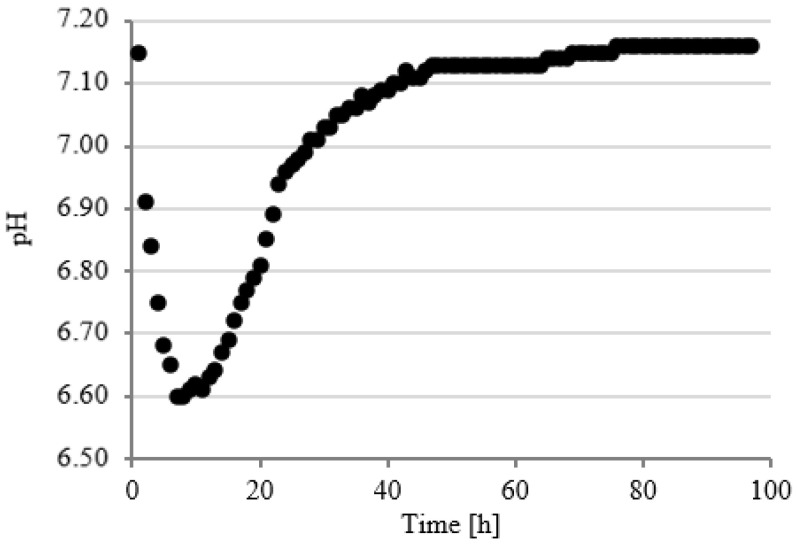
Course of pH changes in series 3.

**Figure 11:**
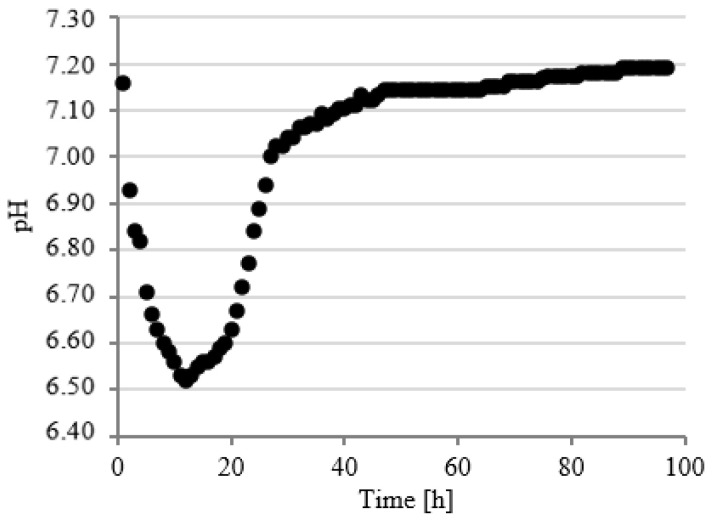
Course of pH changes in series 4.

**Figure 12:**
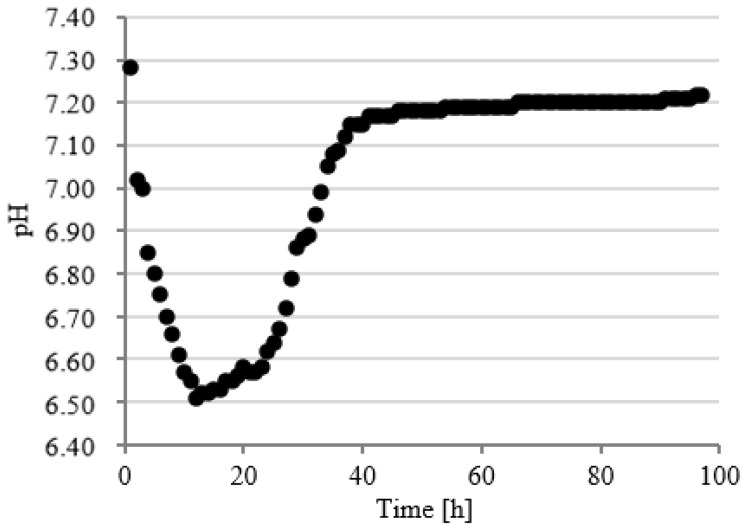
Course of pH changes in series 5.

In series 2 and 3, the pH value dropped significantly faster in the initial stage of fermentation to the values of 6.52 ± 0.13 after 6 h (Figure 9) and 6.60 ± 0.12 after 7 h (Figure 10) of fermentation. These series were also characterized by a more dynamic increase in the pH value. The initial pH value has been reached as early as after 48 h in series 2 (Figure 9) and after 49 h in series 3 (Figure 10). Chavarria et al. (2018) alarmed that methanogenic bacteria were inhibited under conditions with pH < 6.7 [47]. In the present study, the pH was below this value for a short period of time. A similar course of pH changes was observed in series 4 (Figure 11). In contrast, in series 5, the pH stabilized around a minimal value from 12 to 23 h of fermentation and then increased to the initial values, as in the other series (Figure 12). Many authors have concluded that the suppressed efficiencies of wastewater treatment and methane fermentation correlated with the pH drop [25]. In turn, Gannoun et al. (2008) proved that whey acidification with lactic acid and neutralization with lime during AD led to the inhibition of methanogenesis and reduction in process efficiency [24]. These impairments were due to the pH drop from 7.2 to 5.9 triggered by the rapid degradation of sugars to volatile fatty acids [24].

### 3.4. Bacterial Community

The use of multiple variants of acid whey UD had no significant effect on changes in the structure of the anaerobic bacterial community in the digestate (Table 5). Regardless of pretreatment duration, the bacteria turned out to be the prevailing consortium of microorganisms, with their contribution in the population of aerobes ranging from 69 ± 3% in series 2 to 72 ± 2% in series 3. The archaea population accounted for 24 ± 3% in series 2 to 27 ± 1% in series 3 and 5 (Table 5). The observed differences were not statistically significant. The contribution of methanogenes from *Methanosarcinaceae* in the bacterial community ranged from 10 ± 1% to 12 ± 2%, whereas the highest contribution of *Methanosaeta*, i.e., 11 ± 2%, was determined in the control series (series 1). In the series with UD, its contribution ranged from 8 ± 2% to 10 ± 3% (Table 5). Other studies have corroborated the impact of pretreatment on the taxonomic composition of the anaerobic bacterial community. Isa et al. (2022) [48] proved that UD harnessed as a pretreatment technique for palm oil mill effluent (POME) resulted in the most distinct archaea community shift between *Methanosaeta* and *Metanobacterium*. The contribution of *Methanosaeta* increased to 34.4% compared with the 26.6% increase recorded in the case of nondisintegrated POME. In turn, the contribution of *Metanobacterium* decreased to 3.2% from 7.4% in the nonsonicated effluent. The bacteria were still the prevailing microbial domain; however, their contribution in the anaerobic bacterial community decreased to 55% after UD compared with 65% noted in the nondisintegrated effluent [48]. There are also some reports that did not confirm the impact of substrate pretreatment before anaerobic digestion. For instance, Gagliano et al. (2015) [49] observed only that the relative population number of archaea decreased along with the OLR increase in both the sonicated substrate and nondisintegrated one. The use of archaeon-specific FISH probes allowed detecting the presence of long filamentous rods of *Methanosaeta* spp. The latter was found in all digestion phases, highlighting the occurrence of acetotrophic methanogenesis as expected in a mesophilic anaerobic system [49]. 

### 3.5. Removal of Organic Compounds

After the disintegrated whey had been mixed with the inoculum, the initial COD concentration reached 5900 ± 920 mgO_2_/dm^3^ (Table 6). In series 1, the COD removal efficiency reached 68.7 ± 2.2%, ensuring the final concentration of this contaminant at 1850 ± 135 mgO_2_/dm^3^ (Table 6). Harnessing UD for pretreatment facilitated COD biodegradation under anaerobic conditions. Sonication for 300 s performed in series 2 enabled boosting the COD removal efficiency to 71.2 ± 1.7%, which resulted in the final COD concentration of 1700 ± 148 mgO_2_/dm^3^ (Table 6). The highest efficiency of COD biodegradation, approximately 79%, was achieved in series 4 and 5. The concentration of this contaminant in the effluent from the reactors reached 1260 ± 149 mgO_2_/dm^3^ and 1190 ± 160 mgO_2_/dm^3^, respectively (Table 6). Analogous tendencies were observed while monitoring the TOC concentration at the end of the methane fermentation process. The TOC removal efficiency ranged from 71.3 ± 1.5% in series 1 to 80.9 ± 1.6% in series 5, and its concentrations ranged from 1390 ± 128 mg/dm^3^ to 930 ± 47 mg/dm^3^ (Table 6). The highest efficiencies of organic compounds removal were obtained for the biodegradable fraction characterized as BOD_5_. Its initial concentration in the dissolved phase was at 4880 ± 540 mgO_2_/dm^3^. In the most effective variant (i.e., in series 5), it was reduced to 720 ± 65 mgO_2_/dm^3^ at a removal efficiency of 85.3 ± 1.5% (Table 6).

Compared with the present study, a higher maximal COD removal efficiency in methane fermentation, reaching 95%, was achieved upon whey pretreatment by *L. paracasei* at an OLR of 3 gCOD/dm^3^ d [24]. Equally high efficiencies of COD removal, reaching 95.3% [13] and 90% [50], were obtained in a two-stage continuous process of hydrogen bioproduction from whey followed by methane production [13]. The methane fermentation of whey was performed in various types of anaerobic reactors ensuring the efficient removal of organic compounds. For instance, Najafpour et al. (2008) obtained the maximal COD removal (as high as 98%) in an upflow anaerobic-sludge fixed-film (UASFF) reactor at a temperature of 35 °C, HRT of 48 h, and OLR of 25 gCOD/dm^3^∙d [51]. Zieliński et al. (2021) used a microwave-heated multisection hybrid anaerobic reactor (M-SHAR) and achieved over 80% reduction rate of COD at 5 gCOD/dm^3^∙d [25]. A pilot-scale upflow anaerobic filter (UFAF) ensured over 85% removal of COD and 90% removal of BOD_5_ at an OLR of 6 gCOD/dm^3^∙d [52]. In turn, the anaerobic sequencing batch reactor (ASBR) was reported to ensure the removal rates of dissolved COD and BOD_5_ reaching 62% and 75%, respectively, at an HRT of 6 h [53]. Analyses were also conducted to determine the efficiency of anaerobic upflow fixed-film reactors in methane fermentation of whey using various carrier materials, including gravel, charcoal, PVC pieces, brick pieces, and pumice stones, at a temperature of 37 °C [54]. Among them, the charcoal fixed-film reactor showed the best performance when operated at 2 d hydraulic retention times (HRT), achieving maximum COD removal of 81% [54].

### 3.6. Indicators of Methane and Biogas Unit Production

The production of CH_4_ and biogas per unit of removed contaminant proportionally increased with UD time extension. After considering the efficiency of organic compounds removal, the CH_4_ production increased from 0.157 ± 0.02 to 0.267 ± 0.01 dm^3^ CH_4_/g COD_rem._, from 0.184 ± 0.01 to 0.317 ± 0.03 dm^3^ CH_4_/g TOC_rem._, as well as from 0.169 ± 0.02 to 0.299 ± 0.01 dm^3^ CH_4_/g BOD_5 rem._ (Table 7). Regardless of the indicator of the organic compound content in whey, the differences observed in CH_4_ unit production between series 4 and 5 were not statistically significant. Opposite observations were made for the total biogas unit production. In this case, the results of the analysis of the percentage content of CH_4_ in biogas confirmed the significance of the differences between each subsequent experimental series. The unit production of biogas was found to increase from 0.252 ± 0.01 to 0.384 ± 0.03 dm^3^/g COD_rem._, from 0.296 ± 0.02 to 0.455 ± 0.05 dm^3^/g TOC_rem._, as well as from 0.271 ± 0.01 to 0.429 ± 0.05 dm^3^/g BOD_5 rem._ (Table 7). The impact of different UD variants on the biogas unit production from palm oil mill effluent was also proved by Suksaroj et al. (2020) [55]. They applied ultrasounds with powers of 0.27, 0.31, and 0.44 W/cm^3^ for 30, 60, and 90 min, respectively, and achieved the highest CH_4_ production yield, reaching 0.140 dm^3^ CH_4_/g COD_rem._, upon the ultrasonic treatment at 0.44 W/cm^3^ for 90 min [54]. Mischopoulou et al. (2016) [56] formulated similar conclusions from their study into UD of raw molasses wastewater at an ultrasound power of 400 W; ultrasound frequency of 20 kHz; ultrasound wave amplitudes of 90% and 50%; and contact times of 30, 60, 90, and 120 min, during continuous or intermittent reactor work. They demonstrated that the sonication lasting less than 120 min resulted in a low total and unit production of biogas. The highest methane production efficiency was approximately 0.442 dm^3^ CH_4_/gVS [56]. 

### 3.7. Energy Balance

Considering the volume of biogas produced, methane content, and unit energy value of this biogas component, reaching 9.17 Wh/dm^3^, the highest gross energy gain was demonstrated in series 4 and 5, i.e., 9.308 Wh and 9.674 Wh, respectively (Table 8). In the control series without UD, its value barely reached 4.952 Wh (Table 8). The energy consumption for acid whey disintegration was proportional to UD duration and ranged from 1.665 Wh in series 2 to 6.660 Wh in series 5 (Table 8). Considering the energy outputs, the highest net energy gain, reaching 5.763 Wh, was achieved in series 2 (Table 8). In the remaining series, the net energy gain was significantly lower compared with that in the control series and ranged from 4.952 Wh in series 3 to 3.014 Wh in series 5 (Table 8). In the experiment carried out by Mainardis et al. (2019), the surplus of potentially available energy was obtained in the variants with 300 s sonication [27]. All tests conducted at higher sonication energy ranging from 1005.5 to 1387.5 Wh/kgVS and exposure time of 600 s showed a negative final energy balance [27]. UD was observed to generate additional expenditures for the wastewater treatment system [57]; however, it was found a suitable technology to be combined with anaerobic reactors to facilitate the hydrolysis stage and boost the efficiency of complex wastewater treatment systems [58].

### 3.8. Correlations

**Figure 13:**
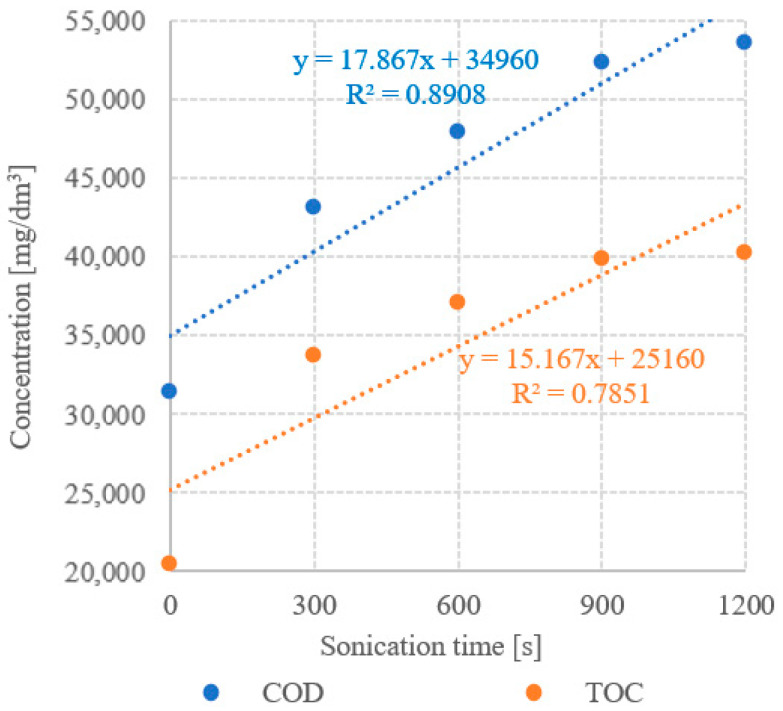
Correlation between UD time and concentration of dissolved organic compounds.

**Figure 14:**
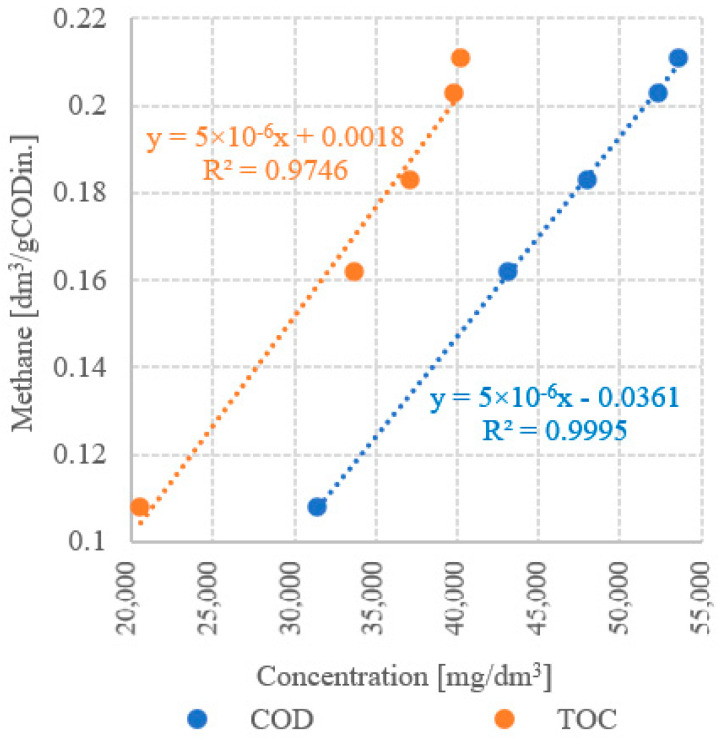
Correlation between concentration of dissolved organic compounds and CH_4_ production.

**Figure 15:**
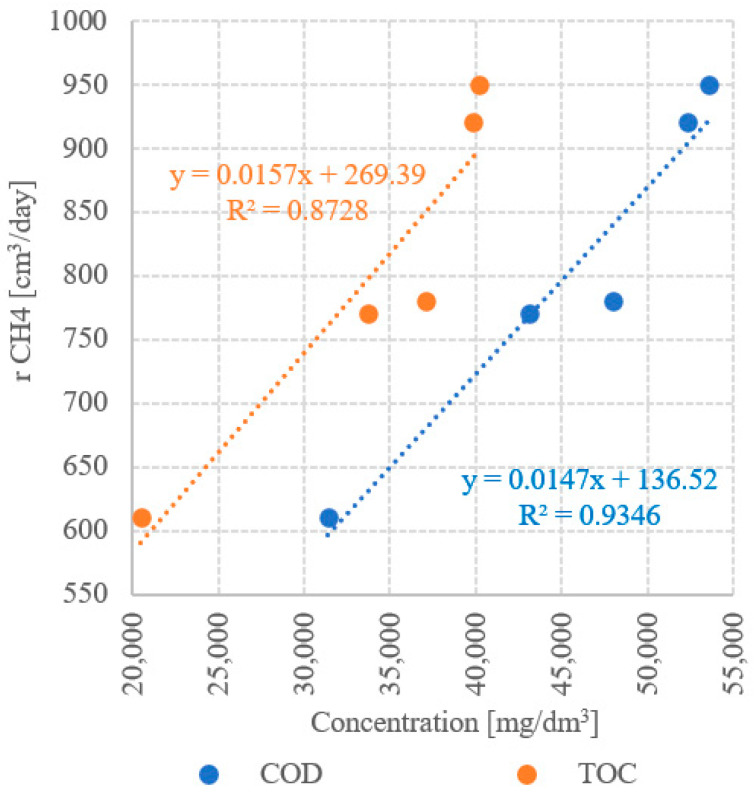
Correlation between concentration of dissolved organic compounds and CH_4_ production rate.

**Figure 16:**
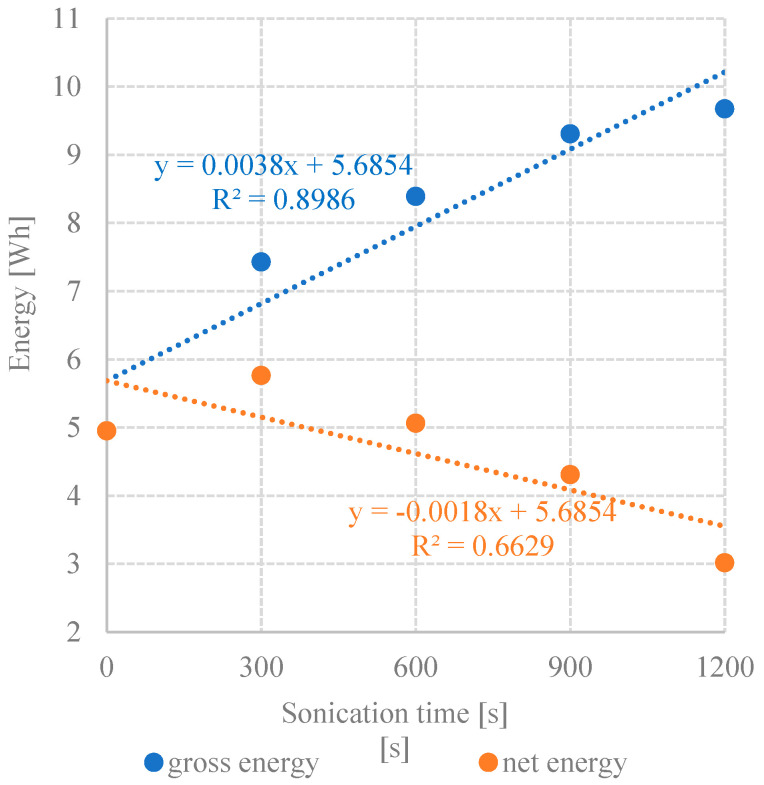
Correlation between UD time and gross and net energy effects.

The results of the analysis of the correlations between UD duration and concentrations of dissolved organic compounds demonstrated strong statistically significant correlations in all technological variants tested. The determination coefficients computed for UD/COD and UD/TOC correlations reached R^2^ = 0.8908 and R^2^ = 0.7851, respectively (Figure 13). Very strong positive correlations were observed between COD and TOC concentrations and CH_4_ yield (Figure 14) and CH_4_ production rate (Figure 15), as indicated by the determination coefficient R^2^ > 0.87. The strongest correlation was found between COD concentration and CH_4_ production (R^2^ = 0.9995) (Figure 14). In contrast, the only negative correlation was determined between UD duration and net energy gain (R^2^ = 0.6629); however, it was not statistically strong (Figure 16). In turn, a strong correlation was noted between UD duration and gross energy gain (R^2^ = 0.8986) (Figure 16).

## 4. Conclusions

The application of UD as a technique for the pretreatment of acid whey had a significant effect on the technological effects of the methane fermentation process. This finding was confirmed by the results of monitoring the concentrations of organic compounds in the dissolved phase and respirometric measurements performed during CH_4_ production. The concentrations of COD and TOC in the liquid phase were proved to depend on UD duration. The greatest difference was obtained between the control series and the series with 300 s UD. The concentration of dissolved COD increased by 11,700 mgO_2_/dm^3^ and that of TOC by 13,200 mg/dm^3^.

The UD variants tested had no significant impact on the structure of the anaerobic bacterial community in the digestate. Regardless of pretreatment duration, the bacteria turned out to be the prevailing bacterial consortium, with about 70% contribution in the anaerobic bacterial community. The archaea population accounted for 24 ± 3% (in series 2) to 27 ± 1% of the anaerobe community.

The effectiveness of the removal of organic compounds from acid whey and the volume of methane produced were found to increase along with extending the time of whey exposure to ultrasounds in the range of 300 to 900 s. The highest technological effects were achieved upon 900 s DU. The CH_4_ production reached 0.203 ± 0.01 dm^3^ CH_4_/gCOD_in._, and its content in the biogas was at 70.9 ± 2.8% The removal rates of organic compounds were at 78.7 ± 2.1% (COD), 80.2 ± 1.3% (TOC), and 84.1 ± 1.6% (BOD_5_). Extending the disintegration to 1200 s had no significant effect on the methane fermentation efficiency. 

The highest net energy gain, reaching 5.763 Wh, was achieved at 300 UD. Extension of the disintegration time had no significant effect on the improvement of the energetic effectiveness of methane fermentation, and the results obtained were comparable with or lower than those determined in the control series. A strong positive correlation was found between the concentration of organic compounds in the dissolved phase and CH_4_ production yield.

## Figures and Tables

**Table 1:** Experimental design.

Series	Sonication Duration [s]	Input Energy [Wh]	Energy Input per Acid Whey Volume (50 mL) [Wh]	Whey Temperature after Sonication [°C]
1	0	-	-	25
2	300	33.3	1.665	40
3	600	66.6	3.330	56.7
4	900	100	5.000	70.7
5	1200	133.2	6.660	79.1

**Table 2:** Characteristics of acid whey solution used in experiments.

Parameter	Unit	Value
COD *	[mgO_2_/dm^3^]	100,000 ± 1200
BOD_5_	[mgO_2_/dm^3^]	87,000 ± 730
TOC	[mg/dm^3^]	82,500 ± 1020
N_tot._	[mg/dm^3^]	4800 ± 260
N–NH_4_	[mg/dm^3^]	140 ± 20
P_tot._	[mg/dm^3^]	1310 ± 130
P–PO_4_	[mg/dm^3^]	390 ± 50
C:N ratio	-	17 ± 1
pH	-	4.9 ± 0.2

COD * = COD_tot_.

**Table 3:** Characteristics of anaerobic sludge (AS) used in experiments.

Parameter	Unit	Value
Dry matter	[%]	3.7 ± 0.3
Organic dry matter	[% d.m.]	69.2 ± 2.8
Mineral dry matter	[% d.m.]	30.4 ± 2.0
N_tot._		38.8 ± 4.7
P_tot._	[mg/g d.m.]	1.7 ± 0.2
TC	[mg/g d.m.]	305.8 ± 13.6
TOC	[mg/g d.m.]	204.8 ± 33.8
C:N ratio	-	9.0 ± 0.2
pH	-	7.38 ± 0.14

**Table 4:** Concentration of organic compounds in the dissolved phase in particular experimental series.

Series	Sonication Time [s]	COD_diss._ [mgO_2_/dm^3^]	TOC_diss._ [mg/dm^3^]
1	0	31,400 ± 482	20,500 ± 399
2	300	43,100 ± 469	33,700 ± 426
3	600	48,000 ± 532	37,100 ± 410
4	900	52,300 ± 645	39,800 ± 583
5	1200	53,600 ± 691	40,200 ± 564

**Table 5:** Microbial taxonomy in AS in particular experimental variants.

Consortium				
Series	Bacteria (EUB338)	Archaea (ARC915)	*Methanosarcinaceae* (MSMX860)	*Methanosaeta* (MX825)
1	70 ± 2	25 ± 2	10 ± 1	11 ± 2
2	69 ± 3	24 ± 3	12 ± 2	9 ± 1
3	72 ± 2	27 ± 1	12 ± 1	8 ± 2
4	70 ± 1	26 ± 3	10 ± 1	10 ± 3
5	69 ± 1	27 ± 1	12 ± 2	9 ± 2

**Table 6:** Efficiency of removal of organic compounds from acid whey in particular experimental series.

Series	Indicator								
	COD			TOC			BOD_5_		
	Initial Concentration [mgO_2_/dm^3^]	Final Concentration [mgO_2_/dm^3^]	Removal Efficiency [%]	Initial Concentration [mg/dm^3^]	Final Concentration [mg/dm^3^]	Removal Efficiency [%]	Initial Concentration [mgO_2_/dm^3^]	Final Concentration [mgO_2_/dm^3^]	Removal Efficiency [%]
1	5900 ± 920	1850 ± 135	68.7 ± 2.2	4850 ± 730	1390 ± 128	71.3 ± 1.5	4880 ± 540	1110 ± 132	77.3 ± 1.8
2		1700 ± 148	71.2 ± 1.7		1300 ± 112	72.9 ± 1.8		980 ± 95	79.9 ± 1.9
3		1500 ± 130	74.6 ± 1.5		1030 ± 69	78.7 ± 1.6		850 ± 86	82.5 ± 2.0
4		1260 ± 149	78.7 ± 2.1		960 ± 72	80.2 ± 1.3		770 ± 88	84.1 ± 1.6
5		1190 ± 160	78.9 ± 1.3		930 ± 47	80.9 ± 1.6		720 ± 65	85.3 ± 1.5

**Table 7:** Methane and biogas production rates by unit of contaminant removed.

**Series**	**dm^3^** **Biogas/g COD_in._**	**dm^3^ Biogas/g COD_rem._**	**CH_4_ Content of Biogas [%]**	**dm^3^ CH_4_/g COD_in._**	**COD Removal Efficiency [%]**	**dm^3^ CH_4_/g COD_rem._**
1	0.173 ± 0.02	0.252 ± 0.01	62.3 ± 2.1	0.108 ± 0.01	68.7 ± 2.2	0.157 ± 0.02
2	0.238 ± 0.01	0.335 ± 0.03	68.9 ± 3.5	0.162 ± 0.01	71.2 ± 1.7	0.228 ± 0.03
3	0.256 ± 0.02	0.343 ± 0.02	71.6 ± 3.2	0.183 ± 0.02	74.6 ± 1.5	0.245 ± 0.03
4	0.286 ± 0.03	0.364 ± 0.04	70.9 ± 2.8	0.203 ± 0.01	78.7 ± 2.1	0.258 ± 0.01
5	0.303 ± 0.02	0.384 ± 0.03	69.7 ± 1.7	0.211 ± 0.02	78.9 ± 1.3	0.267 ± 0.01
**Series**	**dm^3^** **biogas/g TOC_in._**	**dm^3^ biogas/g TOC_rem._**	**CH_4_ content of biogas [%]**	**dm^3^ CH_4_/g TOC_in._**	**TOC removal** **efficiency [%]**	**dm^3^ CH_4_/g TOC_rem._**
1	0.211 ± 0.02	0.296 ± 0.02	62.3 ± 2.1	0.131 ± 0.01	71.3 ± 1.5	0.184 ± 0.01
2	0.290 ± 0.01	0.398 ± 0.04	68.9 ± 3.5	0.197 ± 0.02	72.9 ± 1.8	0.270 ± 0.03
3	0.311 ± 0.02	0.395 ± 0.02	71.6 ± 3.2	0.223 ± 0.01	78.7 ± 1.6	0.283 ± 0.02
4	0.348 ± 0.03	0.434 ± 0.05	70.9 ± 2.8	0.247 ± 0.02	80.2 ± 1.3	0.308 ± 0.03
5	0.368 ± 0.02	0.455 ± 0.05	69.7 ± 1.7	0.257 ± 0.02	80.9 ± 1.6	0.317 ± 0.03
**Series**	**dm^3^ biogas/g BOD_5 in._**	**dm^3^ biogas/g BOD_5 rem._**	**CH_4_ content of biogas [%]**	**dm^3^ CH_4_/g BOD_5 in._**	**BOD_5_ removal** **efficiency [%]**	**dm^3^ CH_4_/g BOD_5 rem._**
1	0.210 ± 0.02	0.271 ± 0.01	62.3 ± 2.1	0.150 ± 0.01	77.3 ± 1.8	0.169 ± 0.02
2	0.288 ± 0.01	0.360 ± 0.02	68.9 ± 3.5	0.225 ± 0.01	79.9 ± 1.9	0.245 ± 0.02
3	0.309 ± 0.02	0.375 ± 0.03	71.6 ± 3.2	0.255 ± 0.02	82.5 ± 2.0	0.268 ± 0.01
4	0.346 ± 0.04	0.412 ± 0.03	70.9 ± 2.8	0.282 ± 0.02	84.1 ± 1.6	0.292 ± 0.02
5	0.366 ± 0.03	0.429 ± 0.05	69.7 ± 1.7	0.294 ± 0.03	85.3 ± 1.5	0.299 ± 0.01

**Table 8:** Energetic efficiency in particular experimental series.

Series	Y_CH4_ [dm^3^ CH_4_/g COD_in._]	Reactor’s Active Volume [dm^3^]	OLR [g/dm^3^]	Total Volume of CH_4_ [dm^3^]	CV_CH4_ [Wh/dm^3^]	E_out_ [Wh]	E_s_ [Wh]	E_net_ [Wh]	E_Uout_ [Wh/gCOD_in._]	E_Us_ [Wh/gCOD_in._]	E_Unet_ [Wh/gCOD_in._]
1	0.108	1.0	5	0.540	9.17	4.952	0.00	4.952	0.990	0.000	0.990
2	0.162			0.810	9.17	7.428	1.665	5.763	1.486	0.333	1.153
3	0.183			0.915	9.17	8.391	3.330	5.061	1.678	0.666	1.012
4	0.203			1.015	9.17	9.308	5.000	4.308	1.862	1.000	0.862
5	0.211			1.055	9.17	9.674	6.660	3.014	1.935	1.332	0.603

## Data Availability

The data presented in this study are available on request from the corresponding author.
